# Reevaluating growing season length controls on net ecosystem production in evergreen conifer forests

**DOI:** 10.1038/s41598-018-36065-0

**Published:** 2018-12-19

**Authors:** David M. Barnard, John F. Knowles, Holly R. Barnard, Michael L. Goulden, Jia Hu, Marcy E. Litvak, Noah P. Molotch

**Affiliations:** 1 Forest and Rangeland Ecosystem Science Center, United States Geological Survey, Boise, ID 83706 United States; 2 Institute of Arctic and Alpine Research, University of Colorado Boulder, Boulder, CO 80309 United States; 3 School of Geography and Development, University of Arizona, Tucson, AZ 85721 United States; 4 Department of Geography, University of Colorado Boulder, Boulder, CO 80309 United States; 5 Department of Earth System Science, University of California, Irvine, CA 92617 United States; 6 School of Natural Resources and the Environment, University of Arizona, Tucson, AZ 85721 United States; 7 Department of Biology, University of New Mexico, Albuquerque, NM 87131 United States; 8 Jet Propulsion Laboratory, Pasadena, CA 91109 United States

## Abstract

Growing season length (GSL) is a key unifying concept in ecology that can be estimated from eddy covariance-derived estimates of net ecosystem production (NEP). Previous studies disagree on how increasing GSLs may affect NEP in evergreen coniferous forests, potentially due to the variety of methods used to quantify GSL from NEP. We calculated GSL and GSL-NEP regressions at eleven evergreen conifer sites across a broad climatic gradient in western North America using three common approaches: (1) variable length (3–7 days) regressions of day of year versus NEP, (2) a smoothed threshold approach, and (3) the carbon uptake period, followed by a new approach of a method-averaged ensemble. The GSL and the GSL-NEP relationship differed among methods, resulting in linear relationships with variable sign, slope, and statistical significance. For all combinations of sites and methods, the GSL explained between 6% and 82% of NEP with *p*-values ranging from 0.45 to < 0.01. These results demonstrate the variability among GSL methods and the importance of selecting an appropriate method to accurately project the ecosystem carbon cycling response to longer growing seasons in the future. To encourage this approach in future studies, we outline a series of best practices for GSL method selection depending on research goals and the annual NEP dynamics of the study site(s). These results contribute to understanding growing season dynamics at ecosystem and continental scales and underscore the potential for methodological variability to influence forecasts of the evergreen conifer forest response to climate variability.

## Introduction

Growing seasons are lengthening across the globe with consequences for ecosystem function including productivity, nutrient cycling, hydrology, and many associated climate feedbacks^1–5^. However, there is little agreement on how to quantify the change in growing season length (GSL) using commonly available field observations. Although many methods exist to quantify metrics of vegetation phenology, calculating GSL from eddy covariance measurements of net ecosystem productivity (NEP) is particularly advantageous due to a large and expanding network of eddy covariance stations^6^, the integrated ecosystem scale of the data, and the simultaneous measurement of biological and meteorological covariates. Yet, substantial uncertainty remains with respect to the impact of lengthening GSLs on NEP (i.e. negative, neutral, or positive relationships), due to the variety of eddy covariance-derived methods used to quantify GSL^7–14^. This lack of standardization highlights the need for an assessment of the variability among methods to quantify GSL, how this variability may differ among ecosystems, and how it may affect inferred ecosystem responses to future climate variability.

Evergreen conifer forests contribute disproportionately to carbon cycling in western North America and other semi-arid areas and have been identified as a research priority due to their potential sensitivity to disturbance^15,16^. Phenological transitions are more fluid in evergreen versus deciduous ecosystems because leaf biochemistry is not constrained to the period between leaf-emergence in spring and senescence in fall^17^, challenging the characterization of a discrete growing season^18,19^. The subsequent potential for rapid shifts between transient dormant states also makes remote sensing methods problematic for estimating phenological phase shifts in conifers. For example, previous studies found that photosynthetic upregulation can precede canopy greening by several weeks^20,21^, suggesting that remote sensing estimates of GSL determined from changes to canopy spectral properties may be biased towards shorter GSLs and later starts to the growing season. In contrast, the eddy covariance technique provides a direct measurement of the integrated ecosystem-scale carbon flux (i.e. NEP) with high temporal resolution^13,14,22,23^. Accordingly, eddy covariance data are commonly used to investigate ecosystem- to global-scale carbon cycling questions, and organized networks of flux towers (e.g., Fluxnet; AmeriFlux) provide an advantageous research platform to characterize patterns of terrestrial carbon cycling through cross-site comparisons^5^. While several methods exist for defining GSL from eddy covariance-derived NEP data, detailed comparisons of these methods are lacking. As a result, it is currently unclear how to interpret among studies that have explored the sensitivity of NEP to growing season length while using different methods^4,8,24,25^.

**Figure 1:**
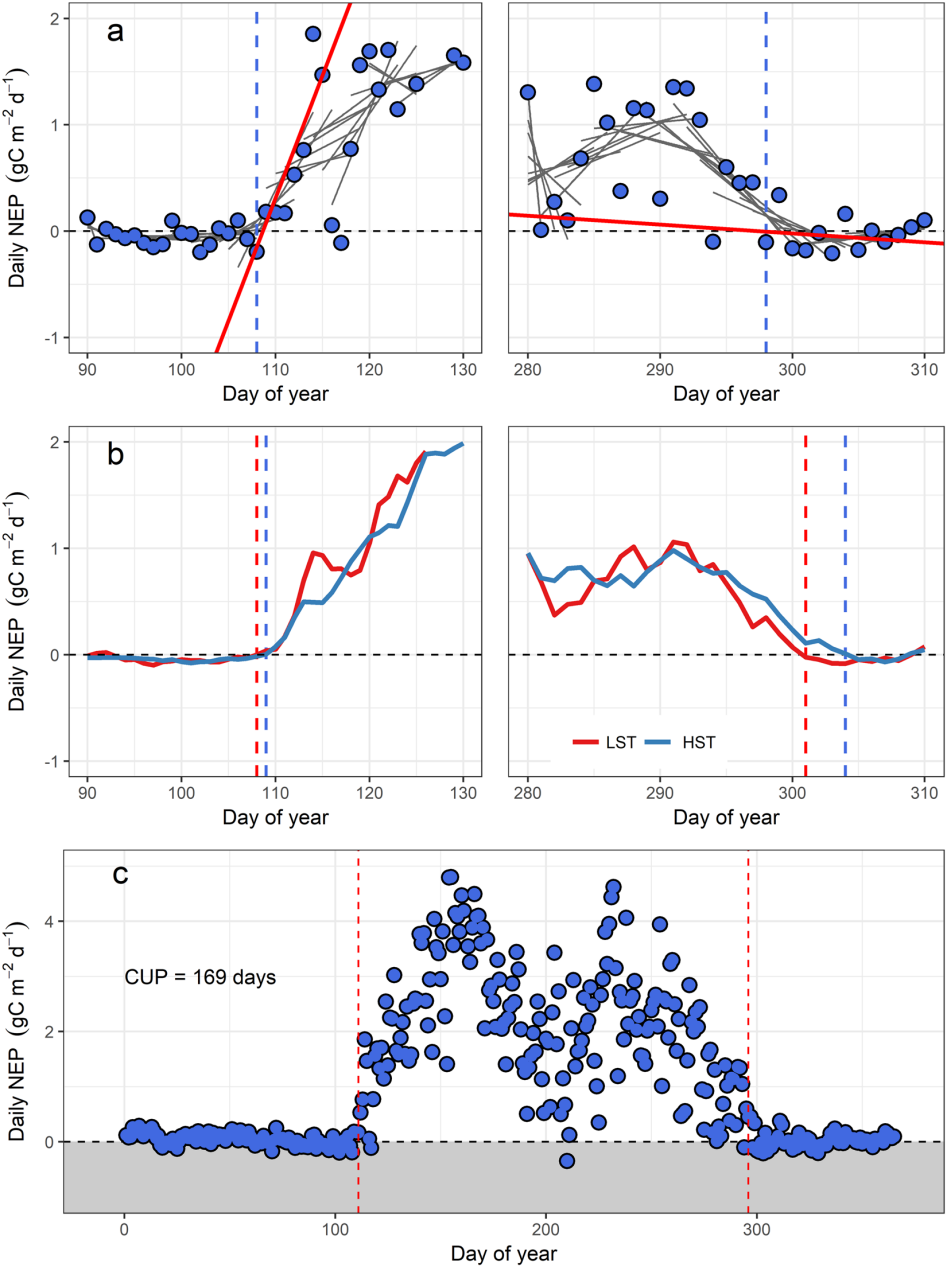
Conceptual illustration of the three methods used to calculate growing season length in this study: (**a**) Variable length regression (VLR) uses linear regression to compute the beginning and end of the growing season as the day with the steepest and shallowest NEP slope, respectively; (**b**) a smoothed-threshold approach uses a 5-day (LST) or a 10-day (HST) moving average to define the start and end of the growing season as the day that the smoothed data cross the zero-NEP threshold (vertical dashed lines represent the beginning and end of the growing season); and (**c**) the carbon uptake period (CUP), where growing season length is determined as the total number of days with daily NEP > 0 (dashed vertical lines represent the mean growing season start and end dates as determined from panels a and b).

This study focused on a subset of the available AmeriFlux data from temperate, subalpine, and boreal evergreen conifer forests in the western United States and Canada that span broad gradients in latitude, air temperature, and precipitation (Table 1). Given that studies in conifer forests often require extensive method- and site-specific tuning to determine GSL^4,13,14,25^, we applied three common groups of generalized methods to facilitate standardized comparison among sites. The first group of methods characterizes the beginning (GS_start_) and end (GS_end_) of the growing season using variable length regressions of day of year versus NEP^24^ (Fig. 1a). The second group fits smoothing functions to daily NEP data where GS_start_ and GS_end_ are determined as the days when a pre-selected NEP threshold is surpassed in spring and fall^10^ (Fig. 1b). The third group does not explicitly characterize a growing season beginning or end date, but instead calculates GSL as the number of days annually that the ecosystem functions as a net carbon sink (i.e. NEP > 0)^10^ (Fig. 1c), potentially including or excluding periods of carbon uptake during winter or stress-induced dormant states during the growing season, respectively^19,26,27^. We focused this analysis on NEP as opposed to gross primary productivity (GPP) because it can be directly measured by the eddy covariance technique and therefore requires no assumptions or statistical estimation of parameters to partition GPP from NEP^25^. We specifically explored the following research questions: (1) what is the variation among three different types of methods to calculate GS_start_, GS_end_, and GSL from eddy covariance data? And, (2) how do these differences influence the subsequent relationship between GSL and NEP within and across ecosystem types and with respect to climatic variability?

**Table 1:** Site characteristics including site name and AmeriFlux site code, principal investigator citation, years of data used in this study, elevation, mean annual air temperature (MAAT), mean total precipitation (MAP), and mean and standard deviation of calculated growing season length (GSL).

Site name and AmeriFlux code	Citation	Years of data used	Site location	Elevation (m)	MAAT (°C)	MAP (mm)	Mean GSL (days)
*Boreal*							
Northern Old Black Spruce (NOBS) (CA-Man)	^9^	1994–2008	MB, Canada	259	−0.5	520	148 ± 40
Poker Flats (US-Prr)	^47^	2010–2014	AK	210	−2.7	275	134 ± 29
*Subalpine*							
GLEES (US-GLE)	^48^	2004–2006	WY	3197	0.4	1200	166 ± 31
Niwot Ridge (US-NR1)	^49^	1998–2014	CO	3050	2.3	800	175 ± 11
Sierra 2700 m (US-SCm)	^50^	2010–2011	CA	2703	4.9	1078	188 ± 19
Valles Subalpine (US-Vcm)	^51^	2007–2010	NM	3030	4.6	646	235 ± 20
*Montane*							
Blodgett (US-Blo)	^52^	1997–2007	CA	1315	11.2	1226	253 ± 33
Flagstaff (US-Fmf)	^53^	2005–2010	AZ	2160	9.5	546	275 ± 19
Metolius (US-Me2)	^54^	2002–2014	OR	1253	7.3	523	276 ± 21
Sierra 2015 m	^50^	2009–2014	CA	2015	10.6	1015	301 ± 55
Valles Montane (US-Vcp)	^55^	2007–2009	NM	2500	6.6	550	277 ± 19

## Results

**Figure 2:**
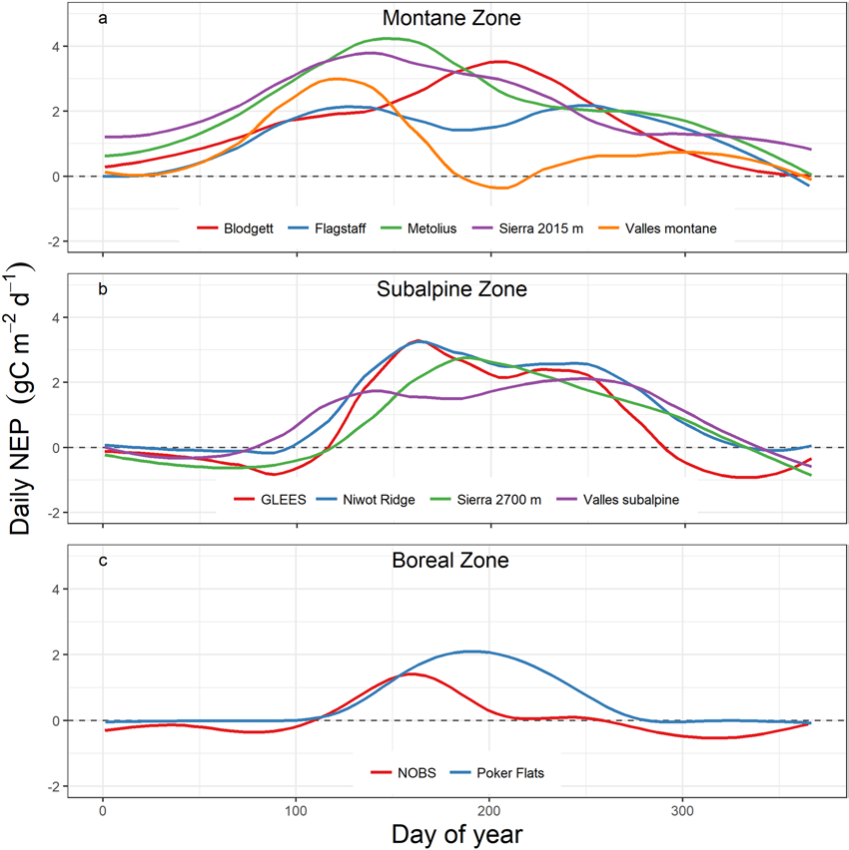
Mean annual dynamics of daily net ecosystem productivity (NEP) among sites and ecotypes. Lines represent a cubic smoothing spline applied to the entire record length at each site.

We used 76 site-years of eddy-covariance tower data, encompassing different record lengths from 11 sites in the AmeriFlux network (Table 1) that represented three globally relevant evergreen conifer ecotypes: temperate montane (Blodgett, Flagstaff, Metolius, Sierra 2015 m, and Valles Montane), subalpine (GLEES, Niwot Ridge, Sierra 2700 m, and Valles Subalpine), and boreal forests (NOBS and Poker Flats). We observed variable annual NEP dynamics among sites that corresponded to zonal and site-specific climatic differences (Fig. 2). In the montane zone, we observed a bimodal NEP pattern at the three driest locations (Valles Montane, Metolius, and Flagstaff), i.e. a mid-summer decrease in NEP followed by increased NEP through the late summer into fall. A similar mid-summer decrease in ecosystem carbon sink strength was characteristic of the subalpine sites except for Sierra 2700 m, but with smaller differences between local maximum and minimum values in early and late summer, respectively. In contrast, the two Mediterranean sites in California (Blodgett and Sierra 2015 m) displayed no corresponding mid-summer decrease and maintained mean daily NEP > 0 for nearly the entire year. In the boreal group, both sites showed distinct carbon source and sink periods, but the period of peak NEP was shorter at the warmer NOBS site relative to the colder Poker Flats site.

**Figure 3:**
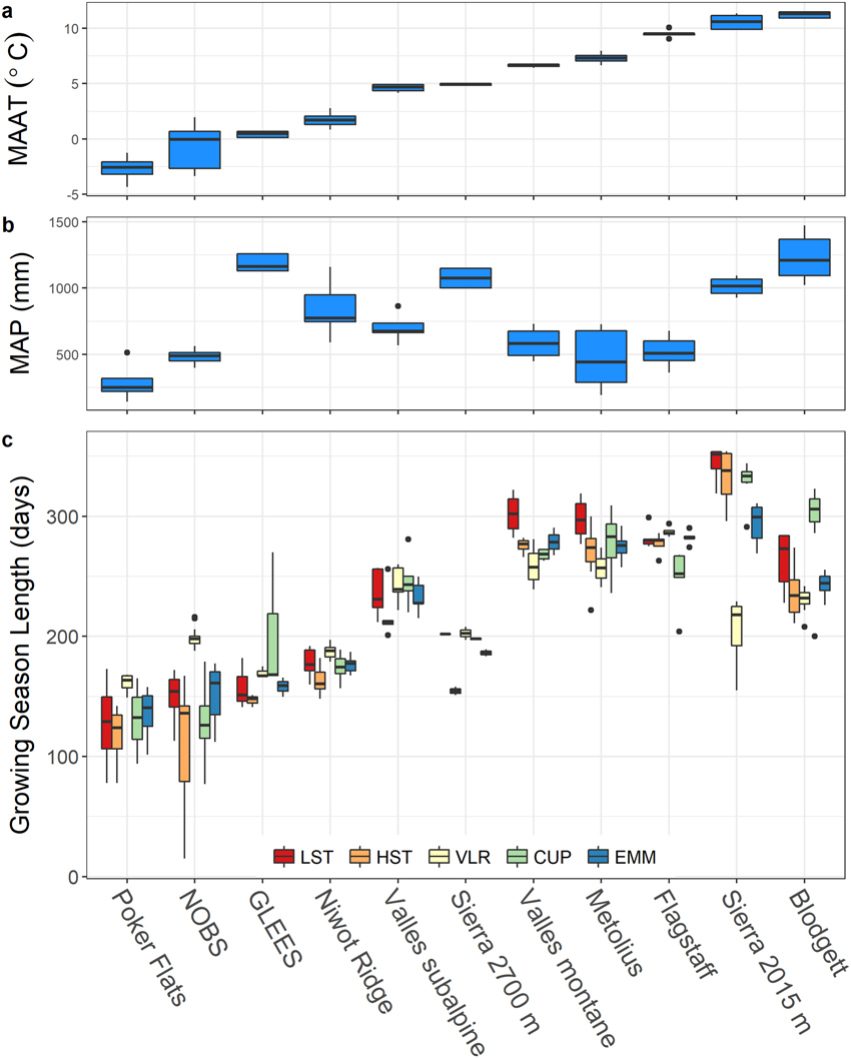
Box and whisker plots of (**a**) Mean annual air temperature (MAAT) (**b**) mean annual precipitation (MAP) and (**c**) plot of interannual variation in growing season length (GSL) among five methods for determining GSL at eleven evergreen conifer sites. Growing season length methodological codes are: low-smooth threshold (LST), high-smooth threshold (HST), variable-length regression (VLR), carbon uptake period (CUP), and the ensemble mean of all methods (EMM).

Irrespective of method, GSL generally increased with increasing mean annual air temperature (Fig. 3). Across sites, the shortest and longest ensemble mean GSLs were 135 and 294 days for Poker Flats and Sierra 2015 m, respectively. The difference in GSL among the three types of methods was significant at all sites (One-way ANOVA, *p* < 0.01) except for GLEES (*p* = 0.23), Poker Flats (*p* = 0.31), and Valles Subalpine (*p* = 0.19). The variability in GSL estimates from the four models (expressed as standard error; se) was generally greatest at the coldest (Poker Flats; se = 12.2 days) and warmest (Sierra 2015 m; se = 7.1 days) sites with Niwot Ridge (se = 1.5 days) having the least variability. There was little variation in standard error among sites with mean annual air temperature (MAAT) between 4 and 10 °C (mean se = 3.2 days). The dispersion of GSL around the mean (coefficient of variation; CV) was greatest at the two boreal sites, Poker Flats and NOBS, with a CV of 20% and 18%, respectively, whereas Sierra 2700 (CV = 2%) and Niwot Ridge (CV = 4%) had the lowest interannual GSL variability. Methodologically, the greatest interannual within-site GSL variability was associated with the carbon uptake period (CUP) method (CV = 11%). The Variable Length Regression (VLR) method resulted in the least interannual within-site GSL variability (CV = 5%). For the smoothing methods, the length of the smoothing window influenced the GSL with five-day smoothing producing a longer GSL than ten-day smoothing by an average of 21 days; this difference was greatest (47 days) at the 2700 m Sierra site and least (3 days) at Flagstaff.

**Figure 4:**
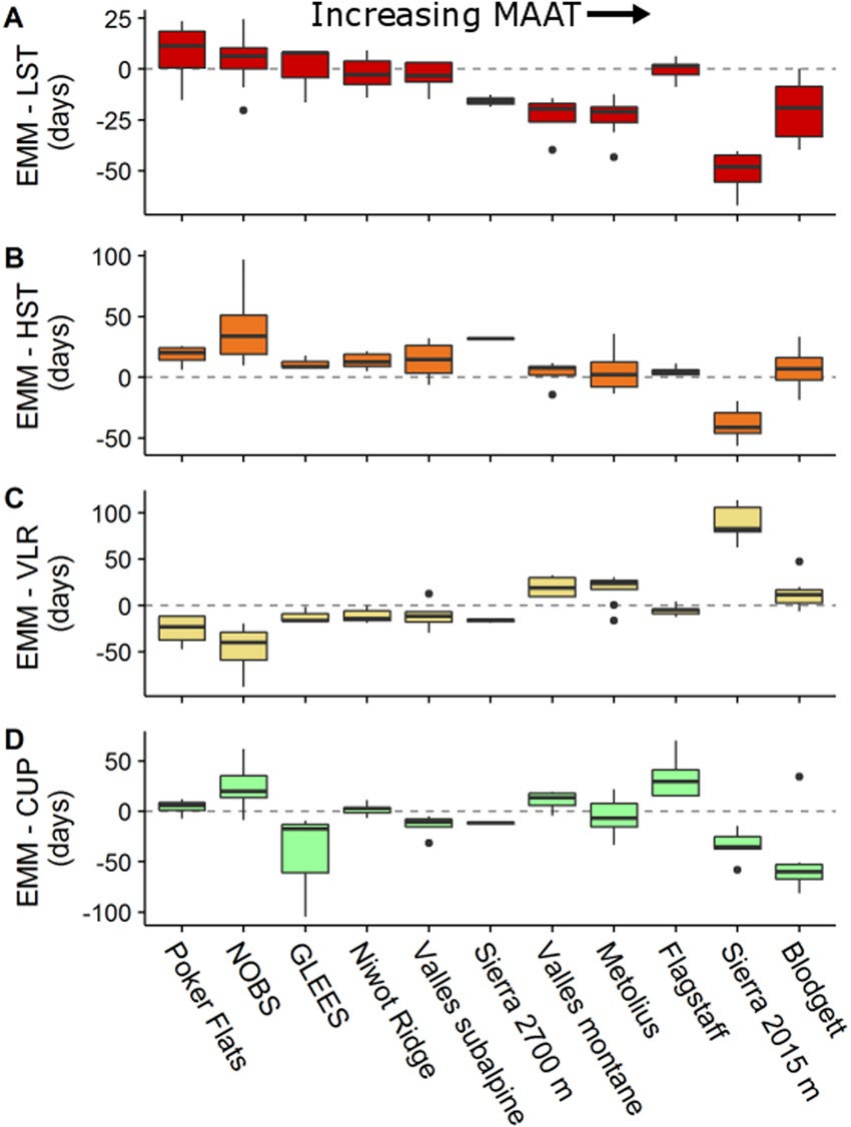
Box and whisker plots for method-specific trends in over- or under-estimation of NEP relative to the ensemble mean follow linear trends when sites are sorted (left-to-right) by increasing mean annual air temperature (MAAT; cf. Table 1). Growing season length method codes are: low-smooth threshold (LST), high-smooth threshold (HST), variable-length regression (VLR), carbon uptake period (CUP), and the ensemble mean of all methods (EMM).

Individual GSL methods were variably biased relative to the response to MAAT represented by the ensemble mean of the different methods (Fig. 4). Both smoothed-threshold methods and the CUP method underestimated GSL compared to the ensemble mean at the colder sites but overestimated the ensemble mean at the warmer sites. While the CUP method underestimated GSL at the coldest site only, the low smoothing threshold (LST) method underestimated the mean GSL at the three coldest sites (Poker Flats, NOBS, and GLEES), and the high smoothing threshold (HST) method underestimated the mean GSL at the nine coldest sites. The VLR method was inversely biased relative to the ensemble mean, overestimating GSL at the colder sites and underestimating GSL at the warmer sites. Regardless of method, the Sierra 2015 site showed an amplified response (i.e. high residual value from the linear fit) compared to the other sites.

**Figure 5:**
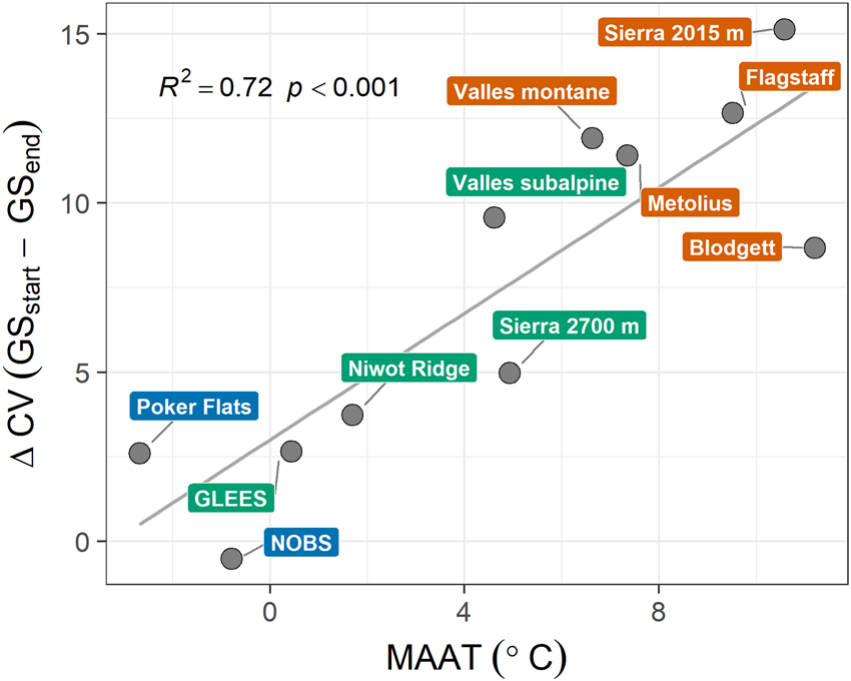
The difference between variability in the start (GS_start_) and end (GS_end_) of the growing season (ΔCV), among the three GSL methods that estimate a GS_start_ and GS_end_ i.e. *n* = 3, as a function of mean annual air temperature (MAAT).

The difference between the temporal variability (ensemble mean of the VLR, LST, and HST methods) associated with the beginning (GS_start_) and end (GS_end_) of the growing season (ΔCV) among sites (2 < *n* < 17) increased linearly with MAAT (Fig. 5). With respect to GS_start_, the Sierra 2015 m site was the most variable (CV = 20%) and Niwot Ridge was the least variable (CV = 5%); the mean CV for GS_start_ across all sites was 11%. With respect to GS_end_, the NOBS site was the most variable (CV = 6%) and Valles Montane was the least variable (CV = 0.7%); the mean CV for GS_end_ across all sites was 2%. The NOBS site was also the only location where inter-annual GS_end_ variability was greater than inter-annual GS_start_ variability. Conversely, GS_start_ was approximately an order of magnitude more variable than GS_end_ at the Valles Montane and Flagstaff sites.

**Figure 6:**
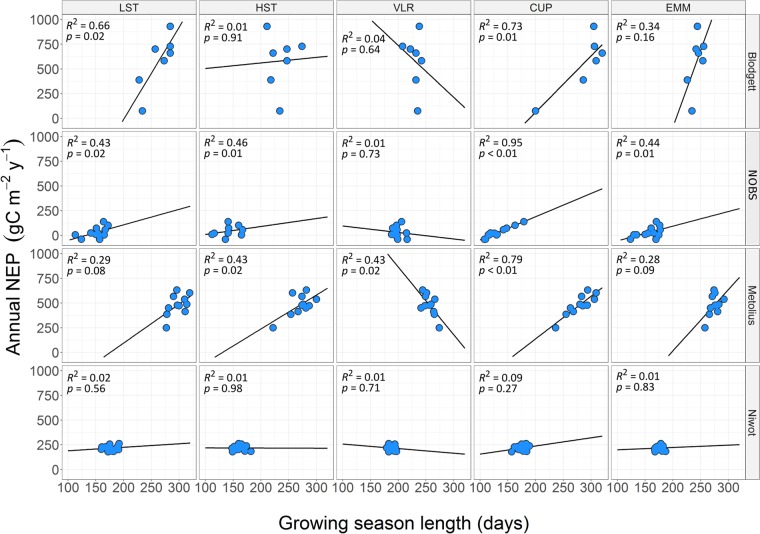
Interannual variability in net ecosystem productivity (NEP) is not consistently explained by different methods to calculate growing season length among the four sites with record lengths greater than 7 years. Methods to determine growing season length include low-smooth threshold (LST), high-smooth threshold (HST), variable-length regression (VLR), carbon uptake period (CUP), and the ensemble mean of all methods (EMM). Each blue point represents the sum of NEP for an individual year.

The ability of GSL to predict NEP was inconsistent among methods and the four AmeriFlux stations with record lengths greater than 7 years (Blodgett, NOBS, Metolius, Niwot Ridge). In certain cases, the among-method variability in GSL was large enough to produce statistically significant (*p* < 0.05) GSL-NEP regression slopes with inverse signs (Fig. 6). Regression coefficients were most consistent among GSL methods at the NOBS and Metolius sites, where all methods except VLR suggested a positive relationship between GSL and NEP. In contrast, Niwot Ridge was the only site where no GSL method significantly explained NEP (cf. Fig. 6 for statistics). Overall, the CUP-GSL method explained the greatest percentage of NEP variance (mean R^2^ = 0.64) and VLR-GSL explained the least (mean R^2^ = 0.12).

## Discussion

Growing season length (GSL) is a common metric in ecosystem ecology used to define vegetation boundaries, constrain biogeophysical models, and/or infer ecosystem responses to future climate shifts^8,10,28,29^. Despite its prevalence in the scientific literature, there is no standardized way to quantify the growing season from eddy covariance data. While previous work has compared methods for determining GSL from air temperature and satellite data^30–32^, to our knowledge this is the first study to systematically contrast commonly used methods to determine GSL from eddy covariance measurements of NEP. By standardizing GSL methods across sites and variable climates, the impact of climate variability on GSL and forest carbon uptake can be isolated and modeled, potentially reducing uncertainty in forecasting future terrestrial carbon dynamics^5,28^. We note that this study was not intended to represent an exhaustive review of all available GSL methods, but rather as an illustration of how the selection of an individual method from a subset of common methods can introduce uncertainty into study results. In this way, we highlight the potential for methodological biases that can affect the magnitude and even the sign of the GSL-NEP relationship when calculating GSL from eddy covariance NEP data.

Calculated GSLs varied among methods at all sites but were most variable at the coldest sites. We therefore highlight that selecting a single GSL method without comparison to other methods has the potential to introduce bias into the interpretation of ecosystem dynamics, especially in high latitude or high altitude systems where vulnerability to a changing climate may be the greatest^2^. We also observed substantial variation in among-method GSL variability at the two warmest sites (Blodgett and Sierra 2015 m), which was likely due to rapid, transient shifts between periods of photosynthetic activity and dormancy in the spring^19^ that can complicate the process of quantifying a discrete beginning of the growing season.

Variability in GSL among methods was principally due to arithmetic differences in how each method identifies and defines shifts in physiological activity. For example, the VLR method selects the date of transition into the growing season by isolating the first day of a multi-day period with the steepest regression slope. However, depending on the rate of transition into the growing season, this point can occur before or after smoothed data crosses the zero-NEP threshold (e.g., Fig. 1a,b). In colder systems, the transition into the growing season occurred later but more rapidly, resulting in VLR-GS_start_ occurring before the moving average crossed the zero-NEP threshold. The opposite was true in warmer systems such as the Sierra sites where an increasing frequency of warm-cold cycles in late-spring produced smoothed data with a more gradual transition into the growing season such that the VLR-GS_start_ generally occurred after the smoothed data had crossed the zero-NEP threshold. Coupled with the general lack of precision in phenological indices, these results support implementations of an ensemble approach that effectively smooths method-specific idiosyncrasies to produce more robust, defensible estimates of GS_start_, GS_end_, and GSL.

The carbon uptake period (CUP) is uniquely unconstrained among the methods in this study by the assumption that all carbon uptake must occur within a discrete period between the beginning and end of the growing season. This creates a particular set of advantages and disadvantages that must be considered prior to its use. For example, our results suggest that the CUP approach is conducive to application in evergreen conifer forests where carbon uptake during periods considered outside of the growing season by other GSL methods can contribute significantly to annual NEP^19^. However, it may not be appropriate for ecosystems that experience warm dry summers (e.g., montane, warm subalpine), and where variability in the maximum daily NEP has been shown to more accurately capture soil or atmospheric drought stress that can reduce carbon fixation such that total respiration produces more carbon than that fixed by photosynthesis (i.e. NEP < 0)^10^. We also emphasize the potential for temporal asynchrony between respiration and gross carbon uptake to affect the CUP method early and late in the growing season^22^. This asynchrony can be especially important in snow-dominated ecosystems, such as several included in this study, where snow cover can introduce a temporal lag into the soil carbon efflux by limiting gaseous diffusion to the atmosphere, potentially skewing temperature-based statistical partitioning estimates of carbon fixation and respiration^33–35^. For these reasons, we focus this study on NEP, as opposed to gross primary production, but acknowledge the benefit of further inquiry into how the constituent components of the ecosystem carbon cycle affect GSL and vice versa.

The high temporal resolution of eddy covariance data is conducive to characterizing ecosystem scale phenology and growing season dynamics given that direct measurements of NEP bypass many errors associated with modeled- and/or satellite-based methods^20,25,36,37^. The co-determination of ecosystem carbon fluxes and GSL from eddy covariance data additionally promotes extrapolation of future conditions from interannual carbon cycling variability, which can supplement modeled representations of ecosystem carbon dynamics in the context of climate variability. However, the methodological disagreement at sites with greater than seven years of data identified by the current study highlights potential risks associated with indiscriminately extrapolating future ecosystem response from eddy covariance GSL and NEP variability. For example, the CUP and HST methods suggested an increase in NEP with increased growing season length at the Metolius site, whereas the VLR method suggested a decrease in NEP under the same conditions. Further, our finding that no method was a significant predictor of the GSL-NEP relationship at Niwot Ridge contrasts previous work that showed a negative GSL-NEP relationship at that location^24^, although this disparity may be affected by recent corrections made to the published Niwot Ridge dataset^38,39^. Regardless, this work identifies the potential for opposing conclusions regarding the NEP response to growing season length to be drawn from the same data, which contributes to lingering uncertainty in carbon cycling projections^28^.

Among ecosystems, the relative timing of GS_start_ and GS_end_ with respect to mean annual air temperature adds context to previous work. For instance, the greater variability in GS_start_ versus GS_end_ was expected given that previous work has shown spring-onset to be more variable than fall-onset^40,41^, and the timing of the winter-to-summer transition is transient in evergreen conifer ecosystems^17^ due to variability of the temperature controls on GS_start_ at both the tree and ecosystem scales^13,14,42,43^. Air temperature anomalies have also been reported to affect GS_end_ variation in temperate and boreal systems, which may be amplified by snow cover effects on albedo and the surface energy balance^35^. However, the enhanced variability of GS_end_ versus GS_start_ at the NOBS site was surprising because boreal carbon assimilation can be limited by day length in autumn to reduce frost damage from delayed dormancy onset^14^. Together with previous studies that have shown greater GS_end_ versus GS_start_ variability in warmer versus colder ecosystems^22,34^, this result could thus signify an important distinction between temperature limitation at colder sites versus water limitation at warmer sites during the autumn. Improved understanding of growing season asymmetries, their effect on annual NEP, and how they vary among ecotypes is essential as spring continues to advance more quickly than fall is delayed, and as warming is amplified at high altitude and high latitude sites^44,45^.

**Figure 7:**
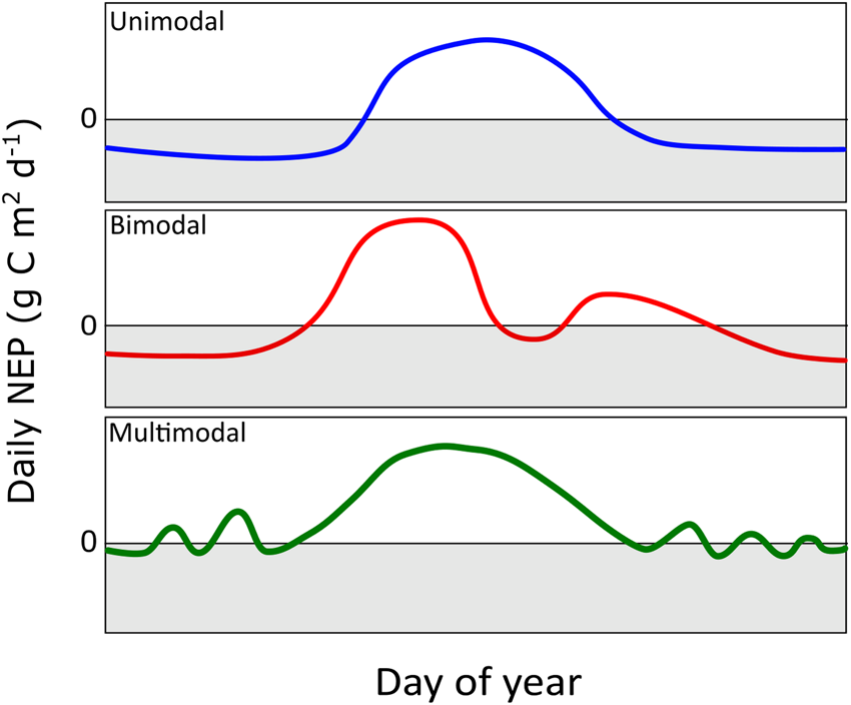
Conceptual representation of the three categories of growing season NEP dynamics identified by this study.

We identified three distinct categories of annual NEP dynamics that are conceptualized in Fig. 7. The first category includes sites that have unimodal annual NEP dynamics, evident from discrete periods of vegetation activity and dormancy, such as cold-dominated boreal and subalpine sites (e.g., NOBS, Poker Flats, GLEES). The second category includes sites that displayed bimodal NEP with defined periods of NEP “dormancy” in mid-summer, potentially due to atmospheric and/or soil moisture stress and generally included warmer and drier montane and subalpine sites (e.g., Niwot Ridge, Valles Subalpine). The third category included sites with multimodal NEP dynamics, such as the Mediterranean montane and subalpine sites (e.g., Sierra 2015 and 2700 m sites), where warm synoptic weather patterns can stimulate significant carbon uptake (NEP > 0) during periods of stress-induced dormancy^19^. This conceptual model illustrates that the efficacy of a chosen GSL method will vary depending on the scope of the study and the system(s) being investigated.

The NEP dynamics identified by this work can be used to guide selection of the most appropriate method for calculating GSL at a given site. For example, the CUP method was the most predictive of NEP, but its usefulness is limited in phenological studies where explicit GS_start_ and GS_end_ dates are needed, and autocorrelation between annual NEP sums and the number of days with net positive carbon fluxes is inevitable. Conversely, methods that define a discrete GSL as the time between GS_start_ and GS_end_ will perform poorly in ecosystems with substantial mid-winter carbon uptake, stress induced dormancy during summer, or where the transition into the growing season is characterized by periods of variable but increasing physiological activity. Given the variability in ecosystem dynamics and how different GSL methods capture phenological transitions differently, we urge future studies to compare multiple methods for determining GSL, and to consider compiling ensemble estimates that can be used to quantify uncertainty (i.e., Fig. 3). We specifically recommend that studies focused on identifying GS_start_ and GS_end_ consider using an ensemble mean of GS_start_ and GS_end_ determined from VLR as well as LST and/or HST methods. These ensembles could be further expanded to include GSL methods not considered in this study but the potential effects of site-specific tuning need to be assessed across multiple sites or regions. Net ecosystem productivity is an integrated measure that represents multiple carbon fixation and utilization processes at various scales and is thus responsive to a myriad of (commonly asymmetric) environmental signals with inconsistent frequency and intensities^26^. Consequently, it is imperative that future investigations continue toward realistic application of GSL-NEP dynamics, especially when seeking to model the phenological response to changing GSL.

## Conclusion

Using 76 site-years of data across a variety of conifer ecotypes, we found substantial variation among methods used to determine the beginning, end, and length of the growing season from daily NEP data. When testing the subsequent influence of GSL on annual sums of NEP, propagation of this variability was sufficient to generate opposite conclusions from different GSL-NEP relationships calculated from a single data set using different methods. The threshold-smoothing and VLR methods tested in this study can define phase shifts but there was evidence for opposite biases among the methodological groups that led to diverging GSLs with increasing mean annual air temperature. Similarly, methods that defined the growing season as the time between GS_start_ and GS_end_ were generally of limited use in ecosystems where the potential for warmer weather patterns during winter and short-term physiological upregulation that contributed significantly to annual sums of NEP. The inconsistent interactions between methodological biases and site-level climate reported herein indicate that an ensemble approach is generally recommended to represent GSL and to characterize the variability resultant from site-specific, methodological, and/or phenological characteristics.

## Methods

Thirty-minute Level 2 processing data files were downloaded directly from the AmeriFlux website (http://ameriflux.lbl.gov/) and NEP measured by tower-based eddy covariance at each site was gap-filled using the REddyProc package^34,46^ in R (version 3.4.4). The selected sites encompassed a wide range of mean annual air temperature (−2.7 to 11.2 °C) and precipitation (275 to 1226 mm) values. We specifically compared three classes of methods to calculate GS_start_, GS_end_, and GSL: (1) Variable-length regression (VLR; Fig. 2a)^24^ computes linear regressions on subsets of total daily NEP from three to seven days in length during spring and fall. The day with the steepest positive slope defines GS_start_ and the day with the shallowest negative slope defines GS_end_. Growing season length is then defined as GS_end_ minus GS_start_. The VLR method was the only method tested by this study to inherently provide a range associated with growing season length, calculated as the variability resultant from the different regressions. (2) The smoothed-threshold method^14^ defines GS_start_ as the day that an interval-averaged NEP crosses and remains above a threshold for a given number of days, with the converse determining GS_end_. We chose zero as the NEP threshold for this study and specifically tested low-smooth (LST; five-day interval) and high-smooth (HST; ten-day interval) thresholds. (3) The carbon uptake period (CUP; Fig. 2c)^8^ defines growing season length as the total number of days in a year that an ecosystem functions as a net carbon sink (i.e., NEP > 0). We also calculated an ensemble mean of all methods as the arithmetic mean GSL from the three previous methods.

## Data Availability

All data are stored in the online AmeriFlux repository available at: http://ameriflux.lbl.gov/.
